# Adolescents with chronic hepatitis C might be good candidates for direct‐acting antiviral therapy

**DOI:** 10.1002/ccr3.5690

**Published:** 2022-04-05

**Authors:** Ken Sato, Yuichi Yamazaki, Yuki Kanayama, Daisuke Uehara, Hiroki Tojima, Takayoshi Suga, Satoru Kakizaki, Naondo Sohara, Norio Horiguchi, Toshio Uraoka

**Affiliations:** ^1^ Department of Gastroenterology and Hepatology Gunma University Graduate School of Medicine Gunma Japan; ^2^ Department of Hepatology Heisei Hidaka Clinic Gunma Japan; ^3^ Department of Gastroenterology National Hospital Organization Takasaki General Medical Center Gunma Japan; ^4^ Sohara Clinic Gunma Japan; ^5^ Department of General Medicine Gunma University Graduate School of Medicine Gunma Japan

**Keywords:** adolescents, chronic hepatitis C, direct‐acting antivirals, Japanese, special population

## Abstract

Three Japanese adolescents with chronic hepatitis C were treated by direct‐acting antivirals (DAAs). No adverse events or laboratory abnormalities were observed during and after DAA therapy, and a sustained virological response was achieved in all cases. The emotional functioning of the patients and their mothers were improved after DAA therapy.

## Key Clinical Message

The efficacy/safety of therapy with direct‐acting antivirals for chronic hepatitis C in Japanese adolescents were favorable and the improvement of emotional functioning of them and their mothers by viral eradication was observed in all cases.

## 1 INTRODUCTION

The global prevalence of hepatitis C virus (HCV) infection among patients aged 1–19 years is estimated to be 0.15%; thus, 3.5 million children suffer from this disease.^1^ In childhood, chronic hepatitis C (CHC) infection is commonly asymptomatic, and cirrhosis and hepatocellular carcinoma rarely develop.^1^ Direct‐acting antiviral (DAA)‐based anti‐HCV therapies have been established as the 1st choice of antiviral agents for adults with CHC worldwide. Sofosbuvir (SOF)/ledipasvir (LDV), elbasvir (EBR) + grazoprevir (GZR), and glecaprevir (GLE)/pibrentasvir (PIB) therapies are the 1st choice of antiviral agents for adults with CHC without previous experience with interferon or DAA therapy according to the most recent Japan Society of Hepatology (JSH) guidelines (ver. 8.0). In addition, the guidelines recommend DAA therapy for CHC in adolescent patients aged 12 years or older, and GLE/PIB therapy is currently approved for these patients in Japan. In Europe, the use of SOF and SOF/LDV is approved for adolescents aged 12 years and over,^2^ but they are not approved for adolescents in Japan. In Part 1 of the DORA study,^3^ GLE/PIB therapy achieved a 100% SVR rate and showed no serious adverse events or clinically significant laboratory abnormalities in adolescent CHC patients aged 12–17 years old. However, this study only included 6 Asian patients, including only 4 Japanese individuals.^3^ Thus, additional evidence of the safety and efficacy of DAA therapy for Japanese adolescents needs to be accumulated.

Here, we successfully treated 3 adolescents with CHC by two kinds of DAA therapy. We obtained informed consent from each patient before treatment.

## 2 CASE PRESENTATION

An 18‐year‐old woman (Case 1) was referred to our hospital for DAA‐based therapy for CHC because she was commuting to a college located in our prefecture. A 16‐year‐old boy (Case 2) was referred to our hospital after consulting with his mother, whose CHC was treated by DAA‐based therapy. A 19‐year‐old man (Case 3) was referred to our department from the Department of Pediatrics, in which he had been followed up in our hospital. The source of HCV transmission was mother‐to‐child transmission in all patients. The HCV genotypes were all 1b. All patients were interferon‐ and DAA‐naive. According to their liver fibrosis markers, the disease severity was diagnosed as chronic hepatitis but not liver cirrhosis. For Case 1, GLE/PIB therapy had not been approved in Japan for adolescent patients with CHC at the time she started treatment. In addition, the previous primary doctor, the patient, and her family agreed to try EBR + GZR therapy due to its excellent efficacy and the paucity of adverse events. Therefore, we measured resistance‐associated substitutions (RASs) of nonstructured protein 5A, that is, Y93H and L31M, which are likely to be resistant to EBR + GZR therapy. Fortunately, neither of them was detected in Case 1. We did not measure RASs in the other Cases. Although their platelet counts were >150,000 cells/μL, abnormalities in serum ALT levels were observed in all cases. The patient characteristics are shown in Table 1.

**TABLE 1:** Patient characteristics, treatments, and their outcomes

Parameters	Case 1	Case 2	Case 3
Patient characteristics			
Age (years)	18	16	19
Sex	Female	Male	Male
Body weight (kg)	56.0	47.5	52.0
BMI (kg/m^2^)	21.3	16.4	17.4
Patient characteristics	Maternal infection	Maternal infection	Maternal infection
HCV genotype	1b	1b	1b
IFN‐based therapy: outcome	None: NA	None: NA	None: NA
DAA‐based therapy: outcome	None: NA	None: NA	None: NA
At the start of therapy			
HCV RNA (log IU/mL)	5.6	5.8	6.6
AST (IU/L)	29	28	24
ALT (IU/L)	32	53	32
WBC (cells/μL)	4590	6470	3150
Hemoglobin (g/dL)	15.4	16.2	15.6
Platelets (cells/μL)	243,000	302,000	180,000
FIB4 index	0.38	0.2	0.45
APRI	0.298	0.231	0.333
M2BPGi (COI)	0.87	0.69	0.94
RAS at baseline	Wild type at Y93 and L31	NA	NA
Severity of liver disease	Chronic hepatitis	Chronic hepatitis	Chronic hepatitis
Treatments and their outcomes			
DAAs	EBR + GZR	GLE/PIB	GLE/PIB
DAA dosage (mg)	50 + 100	300/120	300/120
Weeks of therapy	12	8	8
Concomitant drugs	None	None	None
Achievement of RVR	Yes	Yes	Yes
Adherence to DAAs	100%	100%	100%
Response	SVR24	SVR24	SVR24
Adverse events	None	None	None

Abbreviations: ALT, alanine aminotransferase; APRI, aspartate aminotransferase to platelet ratio index; AST, aspartate aminotransferase; BMI, body mass index; COI, Cutoff index; DAA, direct‐acting antiviral; EBR. elbasvir; FIB4, Fibrosis‐4; GLE, glecaprevir; GZR, grazoprevir; HCV, hepatitis C virus; IFN, interferon; M2BPGi, Mac‐2 binding protein glycosylation isomer; NA, not available; PIB, pibrentasvir; RAS, resistance‐associated substitution; RVR, rapid virological response; SVR24, sustained virological response at posttreatment week 24; WBC, white blood cells.

GLE/PIB therapy was approved in Japan for adolescent patients with CHC just before the start of treatment for Cases 2 and 3. Then, 12 weeks of EBR + GZR therapy and 8 weeks of GLE/PIB therapy were performed for Case 1 and Cases 2 and 3, respectively. The DAA dosage used was the same as that for adults in all cases. The frequency of routine blood sampling was almost the same as that in adults. All cases achieved rapid virological response and sustained virological response at posttreatment 24 (SVR24). There were no adverse events or laboratory abnormalities. After achieving SVR24, emotional functioning such as depression and anxiety was improved in all patients and their mothers by simple hearing investigation (Table 2). Because the female patient was informed by her mother that she was affected with CHC only just one week before physician visit, her emotional functioning was partly impaired.

**TABLE 2:** Change of emotional functioning due to achievement of sustained virological response

	Case 1		Case 2		Case 3	
	Patient	Mother	Patient	Mother	Patient	Mother
Depression						
Feeling down	A	A	A	A	A	A
Loss of energy	NA	A	A	A	A	A
Irritability	NA	A	A	NA	NA	NA
Anxiety						
Nervousness	NA	A	A	A	A	A
Worry	A	A	A	A	A	A
Tension	NA	A	A	NA	A	A

Note

A: improvement; B: no change; C: deterioration; NA: not applicable due to no impairment before administration of direct‐acting antivirals.

The treatments and their outcomes are shown in Table 1.

## 3 DISCUSSION

All 3 cases obtained a rapid virological response and SVR24. No adverse events or laboratory abnormalities were observed. Thus, it is suggested that EBR + GZR and GLE/PIB therapies might be effective and safe even in adolescents.

GLE/PIB therapy was approved in August 2019 in Japan for children over 12 years old with CHC. The most recent JSH guideline (ver. 8.0) recommended therapy should be considered for these populations. EBR + GZR therapy for one patient was started before the approval of GLE/PIB therapy. Our case suggests that EBR + GZR therapy might be useful for these populations. However, the sales of EBR and GZR were discontinued in October 2021 in Japan due to changes in medical needs for these drugs, such as a reduced need for pretreatment measurement of RAS. SOF/LDV therapy is also recommended as a DAA regimen for children and adolescents in American Association for the Study of Liver Diseases and Infectious Diseases Society of America guideline. Thus, we think that indication of SOF/ LDV therapy for these population should be expanded as an alternative to GLE/PIB therapy in Japan in the near future.

The infectious source of all 3 cases was mother‐to‐child transmission, which is in good agreement with a previous study in Japan.^4^ Among HCV‐infected children aged 0–16 years, the frequency of maternal infection was 61%, 92%, 99% in three groups defined by birth year: 1986–1995, 1996–2005, and 2006–2015, respectively.^4^ The genotype of all 3 cases was 1b, which was probably because genotype 1b was predominant in patients in Japan according to the review regarding the molecular‐based epidemiology of HCV, which was published in 2004^5^; however, the HCV genotype 2 was predominant in healthy HCV‐infected children born from 1986 to 2015 in a nationwide survey in Japan.^4^

The most recent JSH guideline (ver. 8.0) states that the indication of antivirals should be decided after taking into consideration that the risk of liver carcinogenesis is low for cases with alanine aminotransferase (ALT) within 30 U/L and platelet counts of 150,000/μL or more. All cases had an ALT exceeding 30 U/L, but the platelet counts were 150,000/μL or more; thus, it can be interpreted that waiting for treatment could also be considered. However, considering previous reports^3^, ^6^ and our case report, the efficacy and safety of GLE/PIB therapy might be favorable even in adolescents and children.

There are several reasons to consider early treatment intervention for adolescents and children with CHC as follows: (1) A previous study showed that 6 (1.8%) of 332 persistently viremic children developed advanced liver disease, 4 of whom were diagnosed with liver cirrhosis in 2–9 years.^7^ Thus, even in adolescents and children, CHC may progress to end‐stage liver disease although the frequency is low.

(2) The incidence of extrahepatic manifestation is less frequent in children with CHC than in adults.^8^ For example, mixed cryoglobulinemia, which is the most common extrahepatic manifestation of hepatitis C and lymphoma,^9^ have not been reported in children. On the contrary, a wide range (8%–65%) of positive results of nonorgan‐specific autoantibodies in children with chronic hepatitis C have been reported, although their clinical significance remains unclear.^10^ A case–control study with 36 children (age 2.7–16.6 years) showed 11.1% subclinical hypothyroidism without anti‐thyroid autoantibodies and 5.6% increased anti‐thyroglobulin levels, and these signs were assumed to be associated with the presence of active HCV infection.^8^ Although the number is small, three children with CHC reportedly developed membranoproliferative glomerulonephritis.^10^ Thus, we should keep in mind that extrahepatic manifestations of HCV infection develop in children as well as adults.

(3) In a case–control study regarding cognitive function in children (3–18 years) with CHC with a normal liver function test, some cognitive functions, such as comprehension, abstract visual reasoning test, quantitative reasoning test, broad memory test, total short‐term memory, and intelligent quotient, were significantly impaired in infected children compared with aged and sex‐matched apparently healthy controls, although the opposite result was obtained with respect to vocabulary function.^11^ The post hoc analysis in a phase 2 study that evaluated the safety and efficacy of SOF and ribavirin therapy in adolescents and children showed that emotional functioning, school functioning, total health‐related quality of life (HRQL) score in baseline parent‐proxy‐reported HRQL scores and school functioning in baseline children’ s reported HRQL scores were significantly lower than the respective healthy youth population scores, and those in adolescents and children with HCV were numerically increased at posttreatment week 24,^12^ which is consistent with our data. Rodrigue et al.^13^ reported that the scaled scores in parent‐proxy‐reported HRQL were not significantly different between children with HCV and the normative sample except for General Health Perceptions and Parent Impact‐Emotional, which were significantly lower in children with HCV than in normative samples. SF‐36 scaled scores for caregivers between the study sample and normative samples were not significantly different, but those for HCV‐infected mothers who transmitted virus to the child were significantly lower in some scaled scores, such as Role‐Physical, General Health, and Role‐Emotional.^13^ On the contrary, the Children's Depression Inventory score in the clinically depressed range was observed in only 2% of children.^13^ Thus, although additional study is required, HCV infection might affect HRQL and cognitive, behavioral, or emotional functioning in children and their caregivers.

However, the timing of treatment intervention should be cautiously considered. A large, multicenter, prospective study of children born to HCV‐infected women in Europe showed that an estimated 21%–25% may have eradicated HCV spontaneously.^14^ When limited to the group in which sustained viral clearance was achieved, serum HCV RNA in 24% of children in the group became undetectable by 3 years of age, and viral clearance was not observed after 5 years of age.^14^ In a multicenter, retrospective/prospective observational study,^7^ HCV clearance was achieved in 27 of 359 (8%) children whose age range was 1–16 years during follow‐up. Out of 27 children, 26 had maternal infections. Spontaneous viral clearance occurred in children with genotype 3 during follow‐up over 5 years.^7^ Genotype 3 was the only independent predictor of spontaneous viral clearance by multivariate Cox analysis.^7^ The investigators suggested that treatment for maternally infected children with HCV genotype 3 could be delayed for up to 5 years.^7^ Thus, we suggest that too early treatment intervention for children, such as prior to 6 years of age, might be avoided.

The limitation of our study is that there are only three case presentations, and the simple hearing investigation regarding emotional functioning was performed after DAA therapies.

## 4 CONCLUSION

Our preliminary results showed that early intervention with DAA therapy for adolescents with CHC might be useful. However, the efficacy and safety of DAA treatment as well as improvement of emotional functioning of adolescents and their caregivers after DAA therapy should be carefully verified in the future because of the very small number of our case series.

## CONFLICTS OF INTEREST

KS received research grants from AbbVie Inc. and MSD K.K. and received lecture fees from AbbVie Inc., MSD K.K., and Gilead Sciences, Inc. YY received a lecture fee from Gilead Sciences, Inc. HT received lecture fees from AbbVie Inc., MSD K.K., and Gilead Sciences, Inc. TS received a research grant from MSD K.K. SK received research grants and lecture fees from AbbVie Inc., MSD K.K., and Gilead Sciences, Inc. NS received a lecture fee from AbbVie Inc. NH received lecture fees from AbbVie Inc., MSD K.K., and Gilead Sciences, Inc. TU received a research grant from AbbVie Inc.

## AUTHOR CONTRIBUTIONS

KS drafted the manuscript. KS, YY, SK, NS, and NH treated the patients and collected the data. YY, YK, DU, HT, and TS proofed the manuscript. YY and TU gave critical advice. KS had given final approval of the version to be published. All authors read and approved the final manuscript.

## ETHICAL APPROVAL

None.

## CONSENT

Written informed consent was obtained from the patient or the patients’ legal guardians for this case series.

## Data Availability

The data are not available for public access because of patient privacy concerns but are available from the corresponding author upon reasonable request.
